# Metabolic Disruption in Osteoporotic Sheep: Evaluating Vitamin D Deficiency and Cortisone Effects via Biochemical Markers

**DOI:** 10.3390/nu17213353

**Published:** 2025-10-24

**Authors:** Gero Knapp, Judith Langenstein, Natali Bauer, Sabine Stötzel, Christian Heiss, Vahid Jahed, Muhammad Alzweiri, Christoph Biehl, Thaqif El Khassawna

**Affiliations:** 1 Experimental Trauma Surgery, Faculty of Medicine, Justus-Liebig-University of Giessen, Aulweg 128, 35392 Giessen, Germany; gero.knapp@chiru.med.uni-giessen.de (G.K.); christian.heiss@chiru.med.uni-giessen.de (C.H.); christoph.biehl@chiru.med.uni-giessen.de (C.B.); 2 Department of Trauma-, Hand- and Reconstructive Surgery, Faculty of Medicine, Justus-Liebig-University of Giessen, Rudolf-Buchheim-Strasse 7, 35392 Giessen, Germany; 3 Department of Veterinary Clinical Sciences, Clinical Pathology and Clinical Pathophysiology, Faculty of Veterinary Medicine, Justus-Liebig-University of Giessen, Frankfurter-Strasse 126, 35392 Giessen, Germany; 4 Biruni University, Istanbul 34015, Türkiye; 5 School of Pharmacy, The University of Jordan, Amman 11942, Jordan

**Keywords:** osteoporosis, sheep model, biochemical markers, bone metabolism, calcium, vitamin, healthy diet

## Abstract

Background/Objectives: We evaluated serum and urinary biomarkers of bone and energy metabolism in an ovine osteoporosis model (Control, OVX, OVXD, OVXDS) at 0/3/8 months (M). Methods: Morning sampling; DXA (ROI ‘abdominal width’) and linear mixed models for repeated measures. Results: Only OVXDS showed severe DXA loss (Z-scores −3.29 at 3 M; −4.86 at 8 M), with ≈20% and ≈30% BMD reductions at 3 M and 8 M versus controls. OVX and OVXD remained within age-expected Z-score ranges at 8 M. At 3 M, OVXDS had hypocalcemia, markedly elevated UFEP, near-zero 25-OH-vitamin-D, and suppressed osteocalcin/NTX (depressed turnover). By 8 M, osteocalcin rose in OVXDS while NTX stayed low, consistent with altered coupling under chronic glucocorticoids and vitamin D deficiency. OVXD showed milder, later changes. Fructosamine and insulin were transiently higher in OVXDS at 3 M; IGF-1 was stable across groups/time. Conclusions: Combined ovariectomy, calcium/vitamin-D-deficient diet, and glucocorticoids produce the clearest biomarker signature and DXA loss. Assay cross-reactivity limited PTH/DKK-1/cathepsin-K measurement in sheep; we summarize DXA outcomes and expand assay limitations and future validation plans.

## 1. Introduction

Osteoporosis is a major global health concern, affecting millions worldwide, particularly in aging populations. The disease is characterized by progressive bone loss, microarchitectural deterioration, and an increased risk of fractures [1]. Osteoporosis-related fractures, especially hip and vertebral fractures, lead to significant morbidity, reduced quality of life, and increased mortality rates [2,3,4].

As the population ages, the global burden of osteoporosis is expected to rise, increasing the need for early detection and effective management strategies to prevent fractures and associated complications. Despite advances in osteoporosis research, diagnosis remains challenging due to the limitations of current diagnostic tools. Dual-energy X-ray absorptiometry (DXA) is widely used to assess bone mineral density (BMD) and predict fracture risk. However, DXA has inherent limitations, including variability in bone density measurements, limited availability in some regions, and an inability to capture dynamic metabolic changes in bone remodeling [5]. Furthermore, osteoporosis is a metabolically active disease, meaning that static imaging methods such as DXA may not fully reflect the ongoing biochemical processes of bone turnover.

The identification of reliable biochemical markers is a promising approach to improve osteoporosis diagnosis and monitoring. Serological biomarkers offer a dynamic assessment of bone metabolism, providing insights into bone formation, resorption, and remodeling activity that cannot be captured by DXA alone [6]. Current biomarkers, such as osteocalcin, bone-specific alkaline phosphatase (BAP), and C-terminal telopeptide (CTX), have been widely studied and are used as indicators of bone turnover [6]. However, their sensitivity and specificity in predicting fracture risk and disease progression remain limited. Recent research has focused on identifying novel biochemical markers that could provide more accurate and specific insights into bone metabolism. Dickkopf-related protein 1 (DKK-1), sclerostin, and cathepsin K have emerged as potential candidates due to their roles in bone remodeling pathways [7].

Despite these advancements, the clinical application of these biomarkers remains limited due to variability in assay sensitivity, species differences, and lack of standardized reference ranges [8]. Further validation studies are necessary to establish biomarker-based diagnostic tools that are reliable across different populations and conditions.

Preclinical research in osteoporosis heavily relies on animal models to investigate disease mechanisms and test potential therapies. Rodent models are commonly used due to their short lifespan and rapid bone turnover; however, their skeletal structure and remodeling dynamics differ significantly from those of humans [9,10,11].

Large animal models, particularly sheep, have become increasingly valuable in osteoporosis research due to their structural and metabolic similarities to human bone [12].

Sheep have comparable cortical-to-trabecular bone ratios, Haversian remodeling, and similar metabolic pathways governing osteoblast and osteoclast activity [12]. These factors make sheep an ideal model for studying age-related bone loss, dietary deficiencies, and hormonal influences on osteoporosis. Additionally, the larger bone size of sheep facilitates biomechanical testing and histological assessments, allowing for a more comprehensive evaluation of bone quality and strength [10].

In this study, we utilized a sheep osteoporosis model to explore the relationship between bone and energy metabolism. The model was designed to simulate hormonal depletion (ovariectomy), dietary deficiencies (calcium and vitamin D), and glucocorticoid exposure, which are major contributors to osteoporosis progression in humans. By examining biochemical markers in serum and urine, we aimed to identify potential non-invasive indicators of osteoporosis that could complement existing diagnostic approaches. Thereby, determine the potential of serological assessments as a complementary diagnostic tool to DXA and assess the effects of dietary deficiency and glucocorticoid exposure on bone metabolism markers. This study hypothesized that dietary deficiency, and glucocorticoid exposure would lead to significant alterations in biochemical markers of bone metabolism, providing evidence that novel biomarkers can enhance osteoporosis diagnosis and monitoring. By addressing the knowledge gap in biomarker-based osteoporosis diagnosis, this study contributes to the ongoing efforts to develop non-invasive, cost-effective, and accessible diagnostic strategies for metabolic bone disorders.

## 2. Materials and Methods

### 2.1. Animal Model

Animal experiments followed the animal welfare act of the National Institute of Health and the guide for care and use of laboratory animals. Experiments also accorded with the national animal welfare guidelines approved by the local regional government and confirmed the German animal protection laws of the district government of Darmstadt (Ref. number V54–19c 20/15–F 31/36). All animal experiments were conducted at the central research facility of Johann Wolfgang Goethe University in Frankfurt am Main.

### 2.2. Experimental Design

This study detected osteoporosis-induced changes in serum and urinary parameters. Changes in other Control loops (such as energy metabolism, electrolyte balance, and body fat composition) can also influence bone metabolism markers and have been considered. The only way to reliably interpret the measured values is by comparison with DXA measurements. This analysis has not been conducted in the present study.

**Figure 1:**
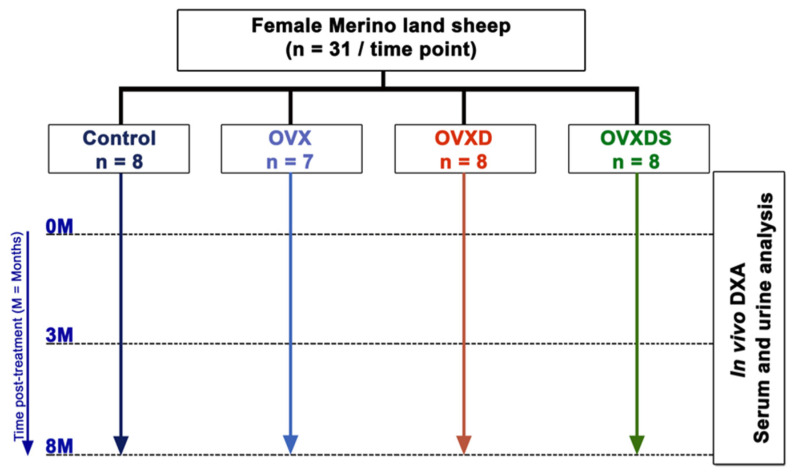
Study Design: this figure depicts the temporal sequence of the animal experiment involving three sampling times (0, 3, and 8 months) across all groups. At the study’s onset, sheep in the OVX, OVXD, and OVXDC groups underwent bilateral ovariectomy, followed by a two-week recovery period. Subsequently, OVXD and OVXDS groups received a deficiency diet, while the OVXDS group also received biweekly glucocorticoid injections until the 8th month endpoint.

In total, 31 of 32 skeletally mature merino sheep were chosen from a shepherd from Wiesbaden, Germany. Sample calculation was performed using G-power analysis with a power of 95% and a *p*-value of 0.05. The inclusion criteria were several different factors (race, no pregnancy, no disease history, no infection, and good general health). Exclusion criteria were animal pregnancy before the experiment (1 animal was excluded). The sheep were stratified randomly and divided into four different treatment groups with an average age of 5.5 years as follows:

(i) Operated not ovariectomized sham group (Control, n = 8);
(ii) Bilaterally ovariectomized group (OVX, n = 7);
(iii) Bilaterally ovariectomized and treated with special calcium- and vitamin-D3-deficient diet for 32 weeks, 2 weeks after ovariectomy (SNIFF Spezialdiäten GmbH, Soest, Germany). (OVXD, n = 8);
(iv) Triple treatment group; in addition to the treatment received in OVXD, this group also received intramuscular steroid suspension every 14 days (320 mg methylprednisolone/sheep) (OVXDS, n = 8) (Figure 1).

### 2.3. Animal Husbandry and Feeding

The animals were kept under the supervision of experienced animal keepers and veterinarians, who guaranteed species-appropriate husbandry. The sheep were kept in small groups of eight animals each. Water and feed were available ad libitum. All sheep received weight controls at the beginning of the study (time 0) and at each subsequent measurement time (3 and 8 months) and were regularly examined by a veterinarian. Furthermore, the sheep were dewormed prophylactically with 1 mL/5 kg KGW Fenbantel (Rintal 2.5% ad us. vet., Bayer AG, Leverkusen, Germany). Serum and urine of the animals were evaluated at Months (M) 0 M, 3 M, and 8 M post-treatment. Blood samples were collected at each measurement time in the morning, considering the circadian rhythm. For this purpose, the jugular vein was punctured with a sterile cannula (20G, Becton Dickinson, Madrid, Spain), and the venous blood was drawn off with a 10 mL syringe (Becton Dickinson, Spain). The sheep were not yet sedated at this time. The whole blood samples were centrifuged for 10 min at a 3460-fold acceleration of gravity (G) (EBA 20, Hettich GmbH and Co., Tuttlingen, Germany), and the serum obtained was aliquoted into 2 mL microtubes with a screw cap (Sarstedt, Nümbrecht, Germany). Urine samples were obtained through the process of catheterization in the ovine subjects. The pre-medicated sheep were stored and catheterized at the respective times of 8 months in the thoracic-abdominal position, and the urine was collected in sterile tubes (10 mL tubes, Sarstedt company, Nümbrecht, Germany). The urine samples were also aliquoted in 2 mL serum tubes with a screw cap (Sarstedt company, Nümbrecht). All samples were placed on dry ice until storage at −80 °C. At the end time point (8 M), blood samples were taken through a central venous catheter (Ein-Lumen-ZVK, 14 G., ARROW Deutschland GmbH, Erdingen, Germany). This was fixed with skin sutures and used to take larger volumes of blood (approx. 50 mL) for injection anesthesia and euthanasia. The animals were euthanized by administering 50 mg/kg pentobarbital (Anestesal, Pfizer, Deutschland GmbH, Berlin, Germany) intravenously while under anesthesia.

### 2.4. Dual-Energy X-Ray Absorptiometry (DXA)

DXA scanning (GE, Lunar Prodigy; enCORE v13.40, Dusseldorf, Germany) was performed under anesthesia. The ROI ‘abdominal width’ was aligned to include L1–L6. We report BMD (g/cm^2^), BMC (g), abdominal fat (%/g), and Z-scores computed as Z = (BMD_group − mean_BMD_controls)/SD_controls, using all 31 sheep at time 0 as the age-matched reference. In human medicine, Z < −1 is considered pathological; we use this threshold to contextualize group changes.

### 2.5. Biochemical and Hormonal Assays

In serum, markers of bone and energy metabolism, insulin-like growth factor 1 (IGF-1), and electrolytes (calcium, magnesium, phosphate), as well as fructosamine and albumin, were determined [6,13,14]. For fructosamine and albumin, the coefficient of variation was <3%. Calcium and magnesium were determined by potentiometry. Phosphate, fractional excretion of phosphate (FEP; hereafter UFEP), fructosamine, albumin, non-esterified fatty acids (NEFA), and creatinine were quantified using enzymatic colorimetric assays. NEFA are an indirect marker for IL-6 and TNF-α activity, which promote osteoclast progenitor cell differentiation [15]. All testing followed the manufacturers’ instructions for commercially available quantitative EIAs/ELISAs (e.g., DRG Instruments GmbH, Marburg, Germany).

Bone turnover assays used for inference were as follows: 25-OH-vitamin D (DRG, competitive EIA; vitamin D–binding protein capture with pre-denaturation; read at 450 nm); bone-specific alkaline phosphatase (BAP; MicroVue EIA; pNPP substrate; read at 405 nm); intact osteocalcin (MicroVue, competitive EIA; read at 405 nm); and amino-terminal telopeptide of type I collagen (NTX; Osteomark ELISA; competitive inhibition; read at 450 nm).

All assays were run on morning samples and processed in batches with internal controls per kit documentation.

### 2.6. Fractional Excretion of Phosphate (UFEP)

UFEP (also referred to as FEP) was calculated as follows: UFEP (%) = (Urine phosphate/Serum phosphate) × (Serum creatinine/Urine creatinine) × 100. Urine creatinine was measured enzymatically (CREA PAP). Urine analytes were diluted as required (typically 1:50) to remain within analytical ranges. (UFEP) was calculated as UFEP = (U_PO_4_ × P_Cr)/(P_PO_4_ × U_Cr). Because UFEP is referenced to the filtered load, it primarily reflects proximal tubular phosphate reabsorption. For interpretation, UFEP was considered together with TmP/GFR (tubular maximum for phosphate normalized to GFR; Walton–Bijvoet approach) to separate tubular transporter effects from changes in filtration [16,17,18]. Where mechanistic attribution is essential, measured or well-validated GFR—for example, iohexol or inulin clearance, or standardized creatinine/cystatin-C–based equations—is recommended to rule out glomerular confounding [19,20].

Creatinine assay and UFEP calculation. Serum and urine creatinine were quantified on the ABX Pentra analyzer using the CREA PAP enzymatic color test (Horiba/Axon Lab); urine was measured after a 1:50 dilution in distilled water. UFEP was calculated as FEP (%) = (U_Phos/P_Phos) × (P_Crea/U_Crea) × 100, where U_Phos and U_Crea are urinary phosphate and creatinine and P_Phos and P_Crea are the corresponding serum concentrations. Assay precision met acceptable performance; example QC showed creatinine CV% ≈ 1% at serum levels and 2.9–5.4% for urinary creatinine across low/high levels

### 2.7. Assay Feasibility Testing (None Inference Data)

Pilot measurements using human ELISAs for parathyroid hormone (PTH), Dickkopf-1 (DKK-1), and cathepsin-K showed inadequate cross-reactivity and/or precision in sheep (values near blanks or non-repeatable). These markers were measured using human ELISA kits in ovine serum. Because species cross-reactivity was poor, we did not calibrate against recombinant ovine standards and did not perform matrix spike-recovery, dilution linearity/parallelism, or bridging to ovine reference materials. Accordingly, these readouts are treated as feasibility only and are reported descriptively without inferential testing or mechanistic interpretation [21,22,23].

### 2.8. Statistical Analysis

Sample-size planning: prior to data collection, an a priori G Power calculation using G*Power 2, opensource software by the Heinrich Heine University Dusseldorf, Germany calculation (F-tests; repeated-measures ANOVA, within–between interaction; α = 0.05; power = 0.80; groups = 4; measurements = 3; assumed correlation among repeated measures = 0.5; nonsphericity correction = 1.0; effect size f = 0.40 based on pilot ovine DXA differences) yielded a target N ≈ 32. One animal was lost, resulting in N = 31, which closely matched the target.

The statistical evaluation was performed using the statistical program SPSS (IBM SPSS Statistics, Version 29, IBM Corp., Armonk, NY, USA). All parameters were analyzed using Linear Mixed Models (LMM) to account for the repeated measures structure of the data and to address the limitations of traditional two-way ANOVA. The LMM approach was chosen because it handles unbalanced data (unequal sample sizes across groups and time points) and incorporates random effects to model subject-specific variability, which was critical given the repeated measurements from the same individuals. In addition, LMM provides flexibility in modeling covariance structures between repeated measures, accommodating potential correlations between time points (e.g., autoregressive or unstructured covariance).

The models included Group (Control, OVX, OVXD, OVXDS), Time Point (0 M, 3 M, 8 M), and their interaction (Group × Time Point) as fixed effects, with Weight and Age as covariates. Random intercepts were included to account for individual variability among subjects. Pairwise comparisons were conducted using Bonferroni adjustment for multiple testing, and the significance level was set at *p* < 0.05.

Residuals from the models were tested for normality using the Anderson–Darling test [24], and Box–Cox transformations were applied where necessary. Compared to traditional two-way ANOVA, the LMM approach allowed for the inclusion of subjects with incomplete data (i.e., missing observations at some time points) and provided robust estimates for fixed effects, even with unequal group sizes. These advantages ensured that the analysis was both comprehensive and statistically reliable. Graphs were finalized using the statistical program Graph Pad Prism (Graph Pad Software Version 5, San Diego, CA, USA).

To avoid non-comparable contrasts, we pre-specified that only time-matched between-group and within-group longitudinal comparisons would be performed and reported.

## 3. Results

In the DXA results, only OVXDS exhibited severe bone loss: Z-scores were −3.29 (3 M) and −4.86 (8 M); BMD medians were ≈20% lower than Control at 3 M and ≈30% lower at 8 M. OVX and OVXD remained within age-expected Z-score ranges at 8 M (−0.29; −0.96). Full DXA tables/plots are provided in the Supplementary File. Per-time point n values are shown in Supplementary File (Table S2).

Unless otherwise stated, all between-group post hoc comparisons were performed within the same time point (Sidak-adjusted); within-group changes were assessed across time. Cross-time, cross-group contrasts were not interpreted.

To comprehensively evaluate changes in variables in homeostasis, all values are reported as [Maximum: Minimum, Mean ± SD] to illustrate inter-group variability; significances are indicated where they occur.

### 3.1. Dietary Deficiency Disrupts Calcium-Phosphate Homeostasis More Severely than Ovariectomy in Osteoporotic Sheep

Both hormonal depletion and nutritional deficiencies contribute to osteoporosis progression, yet their specific effects on calcium-phosphate homeostasis remain poorly understood. To address this, we assessed serum calcium, phosphate, magnesium, and urinary FEP (UFEP) over time.

**Figure 2:**
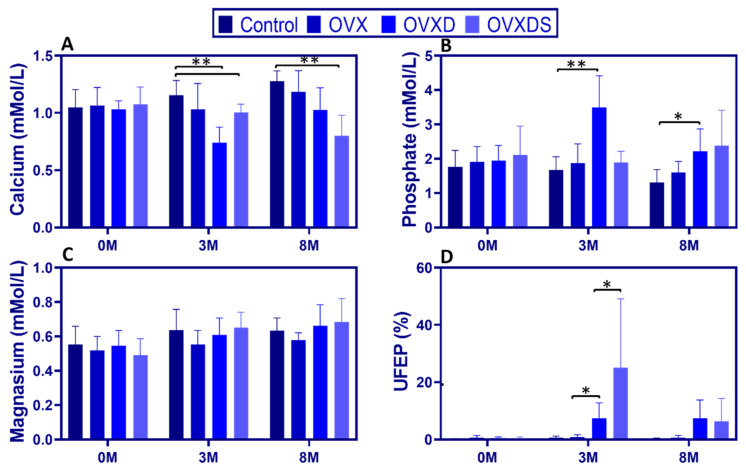
Disrupted calcium and phosphate balance and elevated urinary phosphate excretion in diet-treated groups. (**A**) Serum calcium (mmol/L) declined significantly in OVXD and OVXDS groups at 3 M and remained lower at 8 M. (**B**) Serum phosphate (mmol/L) levels increased significantly in OVXD at 3 M, with elevations persisting at 8 M. (**C**) Serum magnesium (mmol/L) showed no significant differences across groups at any time point. (**D**) Fractional excretion of phosphate (FEP/UFEP%) was markedly higher in OVXD and OVXDS groups at 3 M, with sustained elevation at 8 M. Data are presented as mean ± SD (n = 8/group; * *p* < 0.05, ** *p* < 0.01).

Serum calcium declined significantly in diet-treated groups, with the greatest reductions in OVXD and OVXDS. At 3 M, calcium was lowest in OVXD [0.98:0.61, 0.74 ± 0.14 mmol/L] and OVXDS [1.07:0.83, 1.00 ± 0.07 mmol/L] compared to Control [1.24:0.93, 1.05 ± 0.13 mmol/L], *p* < 0.05. By 8 M, calcium levels in OVXDS remained the lowest [1.01:0.47, 0.81 ± 0.19 mmol/L], with OVXD also significantly reduced [1.31:0.76, 1.03 ± 0.19 mmol/L] compared to controls [1.38:1.11, 1.27 ± 0.09 mmol/L], *p* < 0.01, Figure 2A). In contrast, calcium levels in the Control and OVX groups remained stable.

Diet-treated groups exhibited a significant increase in serum phosphate. At 3 M, phosphate was higher in OVXD vs. Control [4.73:2.43, 3.49 ± 0.93 vs. 2.38:1.21, 1.68 ± 0.38 mmol/L; *p* < 0.001], while OVXDS showed a non-significant trend toward higher values [2.32:1.30, 1.88 ± 0.33 mmol/L]. This pattern persisted at 8 M, with elevated phosphate in OVXD [3.53:1.41, 2.21 ± 0.65 mmol/L] and OVXDS [4.17:1.76, 2.61 ± 0.85 mmol/L] (Figure 2B).

Serum magnesium remained stable across groups. At 3 M, magnesium did not differ between Control [0.84:0.46, 0.64 ± 0.12 mmol/L], OVXD [0.78:0.51, 0.61 ± 0.10 mmol/L], and OVXDS [0.72:0.45, 0.65 ± 0.09 mmol/L], a pattern that persisted (*p* > 0.05; Figure 2C).

UFEP (FEP) increased markedly in diet-treated groups. At 3 M, UFEP was highest in OVXDS [81.49:1.47, 24.97 ± 24.12%] and OVXD [17.11:1.81, 7.35 ± 5.47%] compared to Control [1.82:0.10, 0.65 ± 0.54%], *p* < 0.001. By 8 M, UFEP remained elevated in OVXD [17.07:0.20, 7.33 ± 6.44%] and OVXDS [24.08:0.69, 6.38 ± 7.94%] vs. Control [0.59:0.15, 0.32 ± 0.15%], *p* < 0.05 (Figure 2D). The marked UFEP elevation at 3 M is consistent with increased renal phosphate loss and is compatible with secondary hyperparathyroidism.

These findings indicate that dietary deficiency has a more pronounced impact on calcium-phosphate homeostasis than ovariectomy alone.

### 3.2. Dietary Deficiency Exacerbates Metabolic Disruptions in Bone Turnover Markers and Vitamin D Metabolism

To assess bone turnover and vitamin D metabolism, we measured serum osteocalcin, bone-specific alkaline phosphatase (BAP), amino-terminal telopeptide of type I collagen (NTX), and 25-OH vitamin D over time. Data are presented as [Max:Min, Mean ± SD].

**Figure 3:**
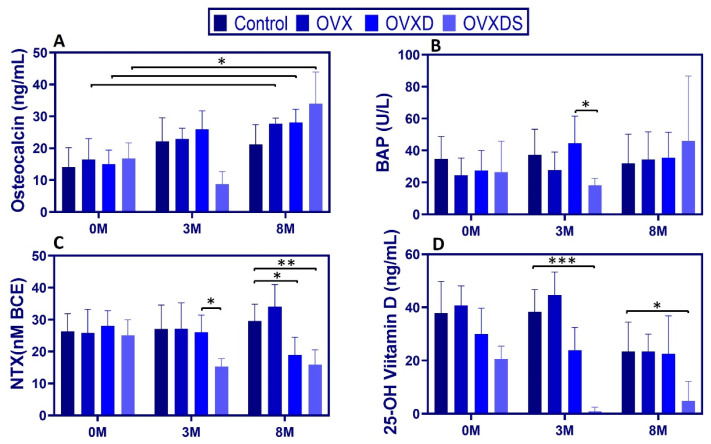
Dietary deficiency exacerbates disruptions in bone turnover markers and vitamin D metabolism. (**A**) OC increased in diet-treated groups over time, with the lowest OVXDS values at 3 M (significantly below Control and OVX; Sidak *p* < 0.01) and the highest at 8 M. (**B**) BAP was lower in OVXDS vs. OVXD at 3 M, with no differences by 8 M. (**C**) NTX was reduced in OVXD and OVXDS by 8 M. (**D**) 25-OH vitamin D was markedly reduced in diet-treated groups, with the greatest decline in OVXDS at 3 M and sustained reduction at 8 M. Data are mean ± SD (n = 8/group; * *p* < 0.05, ** *p* < 0.01, *** *p* < 0.001).

Osteocalcin (OC). At 3 M, OC was significantly lower in OVXDS vs. Control and vs. OVX (Sidak *p* < 0.01) and higher in OVXD vs. OVXDS [29.59:12.75, 23.80 ± 5.84 ng/mL; *p* < 0.05]. By 8 M, OC reached its highest levels in OVXDS [40.16:12.45, 31.39 ± 9.93 ng/mL; *p* < 0.01], while Control remained stable [24.81:8.25, 19.11 ± 6.20 ng/mL] (Figure 3A).

BAP. At 3 M, BAP was significantly lower in OVXDS vs. OVXD [24.10:10.50, 16.49 ± 4.40 vs. 74.90:22.80, 42.90 ± 17.02 U/L; *p* < 0.01]. By 8 M, BAP no longer differed significantly across groups (Figure 3B). NTX. At 3 M, NTX in Control was [37.77:14.83, 27.05 ± 7.54 nM BCE], compared to [36.40:20.41, 26.16 ± 5.41 nM BCE] in OVXD and [24.83:11.96, 16.21 ± 4.10 nM BCE] in OVXDS (*p* < 0.05). At 8 M, OVXD [37.91:11.81, 21.28 ± 8.47 nM BCE] and OVXDS [23.18:10.79, 15.89 ± 4.67 nM BCE] were significantly lower than Control [47.93:24.99, 34.78 ± 7.16 nM BCE] (*p* < 0.01 and *p* < 0.001, respectively; Figure 3C). OVXD was also lower than OVX at 8 M (*p* < 0.05).

25-OH vitamin D. Vitamin D deficiency was evident in diet-treated groups from the outset. At 0 M, 25-OH vitamin D was already lower in OVXD [49.64:15.73, 30.00 ± 9.68 ng/mL] and OVXDS [31.01:16.08, 20.66 ± 4.74 ng/mL] vs. Control [59.63:22.29, 37.94 ± 11.80 ng/mL], *p* < 0.01. Because baseline values differed, we report baselines explicitly and interpret 3 M/8 M differences with caution; a sensitivity ANCOVA adjusting for baseline 25-OH vitamin D is provided in Supplementary File (Table S3). By 3 M, 25-OH vitamin D in OVXDS had decreased nearly 38-fold from baseline and remained significantly lower than Control (*p* < 0.001). This deficiency persisted at 8 M, with OVXDS still ≈10-fold lower than Control [16.72:0.00, 4.84 ± 7.33 vs. 33.49:0.00, 23.38 ± 11.12 ng/mL; *p* < 0.01] (Figure 3D). These findings demonstrate that dietary deficiencies exacerbate bone turnover dysregulation and vitamin D depletion.

Furthermore, Serum creatinine remained within physiological ranges across groups and timepoints, with no between-group differences at 8 M and expected variability in spot samples at 3 M (Table S4). These data argue against a primary glomerular contribution to the phosphate changes; therefore, the elevated UFEP we observed is most consistent with reduced proximal tubular phosphate reabsorption rather than changes in filtration. UFEP/FEP values were computed from paired phosphate and creatinine measurements for each sample.

### 3.3. Dietary Deficiency Disrupts Energy Metabolism and IGF-1 Signaling in Osteoporotic Sheep

**Figure 4:**
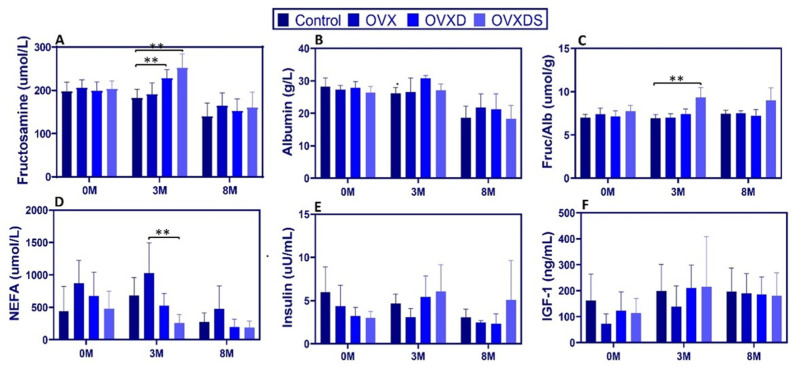
Dietary deficiency disrupts energy metabolism and IGF-1 signaling in osteoporotic sheep. (**A**) Fructosamine was elevated in OVXD and OVXDS at 3 M. (**B**) No significant changes were observed between the groups at each time point. (**C**) The FA ratio was higher in OVXDS at 3 M and remained higher at 8 M versus the Control. (**D**) NEFA was elevated in OVX at 0 M and was lower in OVXDS vs. OVX at 3 M. (**E**) Insulin was transiently higher in OVXDS at 3 M; no group differences at 8 M. (**F**) IGF-1 increased gradually over time without group differences. Data are mean ± SD (n = 8/group; ** *p* < 0.01).

To investigate energy metabolism and IGF-1 signaling, we measured serum fructosamine, albumin, fructosamine-to-albumin ratio (FA ratio), NEFA, insulin, and IGF-1 over time. Data are presented as [Max:Min, Mean ± SD]. Fructosamine increased significantly at 3 M in diet-treated groups. At 3 M, levels were higher in OVXD [258.00:203.00, 227.88 ± 19.49 µmol/L] and OVXDS [313.00:220.00, 251.62 ± 32.62 µmol/L] vs. Control [212.00:146.00, 181.62 ± 20.91 µmol/L], *p* < 0.0001 (Figure 4A). No significant differences were observed at 0 M or 8 M.

Albumin non-significantly declined over time, with hypoalbuminemia observed across all groups by 8 M. At 3 M, the FA ratio was elevated in OVXDS [11.2:5.9, 8.32 ± 1.38 µmol/g] vs. Control [7.4:3.5, 5.24 ± 1.13 µmol/g], *p* < 0.01; this persisted at 8 M (*p* < 0.01) (Figure 4C). At baseline, NEFA were non-significantly higher in OVX [1432.30:237.80, 676.20 ± 362.53 µmol/L] vs. Control [933.60:93.50, 443.65 ± 379.64 µmol/L], *p* > 0.05. At 3 M, NEFA were lower in OVXDS vs. OVX [506.00:114.00, 261.36 ± 126.35 vs. 1597.80:264.30, 1016.41 ± 504.84 µmol/L; *p* < 0.05] (Figure 4D). At 8 M, NEFA declined in all groups, with no differences. Insulin showed a transient elevation in OVXDS vs. OVX at 3 M [18.97:2.55, 7.83 ± 5.43 vs. 7.19:2.01, 3.69 ± 1.79 µIU/mL; *p* < 0.01] (Figure 4E). By 8 M, no group differences were detected, though relative hyperinsulinemia persisted in OVXDS vs. Control. These measurements were obtained in the fed morning state. IGF-1 remained stable across groups; levels increased gradually over time without group differences (*p* > 0.05; Figure 4F). Exploratory biomarkers. DKK-1 and sclerostin ELISAs were exploratory and limited by species-specific assay performance. DKK-1 values were low but measurable (3 M: 5.19 ± 0.25 pmol/L; 8 M: 4.70 ± 0.25 pmol/L; CV 4.88–5.33%) and are presented descriptively. Sclerostin showed high variability and inadequate cross-reactivity; therefore, sclerostin results were excluded from inferential analysis. Exploratory biomarker plots are shown descriptively in Supplementary File (Table S5).

## 4. Discussion

This study provides new insights into the metabolic consequences of dietary deficiency and glucocorticoid exposure in osteoporosis using a large animal model. The findings demonstrate that calcium–phosphate homeostasis, bone turnover markers, and vitamin D metabolism are significantly altered in response to nutritional and hormonal disturbances, emphasizing the crucial central role of diet and systemic metabolism in osteoporosis progression. The data suggest that serological biomarkers could complement traditional DXA-based diagnostics, offering a functional perspective on disease progression activity that may improve early detection and sharpen management monitoring. The concise DXA summary shows severe loss confined to OVXDS (Z-scores −3.29 at 3 M and −4.86 at 8 M), whereas OVX and OVXD remained within the age-expected range at 8 M (see Supplementary Materials—DXA Results). Unless otherwise noted, between-group comparisons are interpreted within the same time points, and baseline differences (notably for 25-OH-vitamin-D) are reported explicitly. A primary objective of this study was to evaluate how dietary deficiency impacts bone metabolism compared relative to hormonal depletion alone. The results indicate that nutritional deficiencies. Nutritional deficits, particularly in calcium and vitamin D, exerted a more pronounced effect on bone metabolic disturbances than ovariectomy alone. This was, evidenced by marked reductions in reduced serum calcium levels and a corresponding increase in urinary phosphate excretion (FEP) in diet-treated groups, which suggests consistency with a compensatory parathyroid hormone (PTH) response. Although direct PTH measurements were not available due to species-specific assay limitations, the metabolic alterations observed align with previous findings in secondary hyperparathyroidism models, reinforcing the idea that chronic nutritional deficiency can drive osteoporosis progression independently of hormonal ovarian hormone depletion [25,26].

Consistent with this interpretation, the urinary fractional excretion of phosphate (UFEP; FEP) was markedly elevated at 3 M in OVXDS (and increased in OVXD), compatible with renal phosphate loss under secondary hyperparathyroidism. Beyond this endocrine explanation, PTH-independent mechanisms plausibly account for the UFEP rise. FGF-23/α-Klotho signaling acts directly on proximal tubules to down-regulate NaPi-IIa/IIc (SLC34A1/SLC34A3), thereby increasing phosphaturia; in parallel, FGF-23 suppresses CYP27B1 (1α-hydroxylase) and lowers calcitriol, further promoting phosphate wasting—both mechanisms independent of PTH [27,28,29,30]. In keeping with renal physiology and clinical guidance, UFEP is best interpreted alongside TmP/GFR; when both indicate reduced tubular reabsorption, a tubular origin is likely, whereas incorporation of measured or well-validated GFR helps exclude glomerular confounding [16,18,19,20]. Future work will quantify FGF-23, α-Klotho, calcitriol, and GFR in parallel to partition endocrine from tubular drivers of phosphaturia in this ovine model. Importantly, serum creatinine was comparable across groups at all time points (Table S4), reinforcing that the elevated UFEP reflects reduced tubular reabsorption rather than a glomerular filtration effect.

This UFEP pattern is consistent with the broader calcium–phosphate phenotype we observed. A particularly striking observation was the strong correlation between vitamin D deficiency and secondary hyperparathyroidism in diet-treated groups. OVXD and OVXDS exhibited the most pronounced reductions in serum calcium, accompanied by marked increases in urinary phosphate excretion (prominently in OVXDS and present in OVXD), suggesting elevated PTH activity as a compensatory mechanism. These findings align with human osteoporosis literature in which chronic vitamin D deficiency drives secondary hyperparathyroidism, exacerbating bone resorption and fracture risk [31,32,33]. Given the prevalence of vitamin D deficiency in aging populations, early nutritional intervention is warranted. Because baseline 25-OH-vitamin-D differed between groups, baseline values are reported explicitly, and 3 M/8 M differences are interpreted within time points; a sensitivity analysis (ANCOVA with baseline as covariate) is outlined in Section 2 and Supplementary Materials. Given the widespread prevalence of vitamin D deficiency in aging populations, these findings emphasize the importance of early nutritional intervention to prevent metabolic bone disease progression. Because baseline 25-OH-vitamin-D differed between groups, we report baseline values explicitly and interpret 3 M/8 M differences within time points; a sensitivity analysis (ANCOVA with baseline as covariate) is outlined in Section 2 and Supplementary Materials.

Glucocorticoid administration further exacerbated suppression of bone turnover suppression, as evidenced by significantly reduced levels of osteocalcin (over time) and BAP (at 3 M) in the OVXDS group. These findings are consistent with previous reports demonstrating that glucocorticoids impair osteoblast function and can reduce osteoclast number/function while impairing mineralization, ultimately leading to collectively reducing bone formation capacity and increasing skeletal fragility [34,35]. In our model, NTX decreased in OVXD/OVXDS by 8 M, while osteocalcin rose in OVXDS, suggesting uncoupled turnover (late OC↑ with persistently low resorption) rather than coordinated increases in both formation and resorption.

We also addressed analytical validity for exploratory biomarkers. PTH, DKK-1, and sclerostin were measured as feasibility assays to scope effect direction, but current data are non-quantitative due to methodological constraints. The human ELISAs used in ovine serum lacked demonstrated cross-reactivity, were not calibrated with recombinant ovine standards, and were not supported by matrix spike-recovery, dilution linearity/parallelism, or bridging experiments; results are therefore presented descriptively and excluded from inference [21,22,23]. Going forward, we will (i) secure or develop ovine-specific antibodies and calibrators; (ii) execute fit-for-purpose validation (cross-reactivity, spike-recovery, dilution linearity/parallelism, precision, stability); and (iii) implement orthogonal quantification by targeted LC–MS/MS of proteotypic peptides with stable-isotope internal standards, optionally preceded by immunoaffinity enrichment (SISCAPA/immuno-MRM), to confirm concentrations independently of immunoassay epitope effects [36,37,38,39]. This framework will enable quantitative, species-appropriate readouts and strengthen translational reliability for these markers in sheep.

The link between osteoporosis and osteoporosis is increasingly recognized as systemic metabolism has gained increasing attention in recent years. Our findings indicate that metabolic disease. Here, dietary deficiency and glucocorticoid exposure also influence energy metabolism, as indicated by elevated fructosamine levels at 3 M in OVXDS (at 3 M), suggestive of, consistent with, altered glucose metabolism and handling/insulin resistance. Serum insulin showed a transient elevation that rose transiently at 3 M (OVXDS vs. OVX), normalizing) and normalized by 8 M; because all measurements were obtained sampling in the fed morning state, these findings should be interpreted with caution, and warrant fasting/or dynamic testing is warranted in follow-up.

In contrast to human and rodent data, where glucocorticoids often lower circulating IGF-1, ruminants display regulatory features that can buffer total IGF-1 when nutrition and hepatic function are preserved. In cattle and sheep, circulating IGF-1 closely tracks energy balance and hepatic growth hormone receptor (GHR) signaling, such that maintained metabolic status sustains IGF-1 output despite physiological or pharmacologic stress [40,41,42]. Moreover, continuous rumen fermentation supplies volatile fatty acids and moderates meal-to-meal fluctuations, attenuating the acute feed–fast oscillations that destabilize the GH→–IGF-1 axis in monogastrics [30]. These ruminant-specific features provide a mechanistic rationale for the observed stability of total IGF-1 across treatments in our ovine model.

To probe this buffering directly, future work should (i) standardize sampling state (fasted vs. post-prandial) and timing; (ii) quantify IGF-binding proteins (IGFBP-1/IGFBP-3) and the acid-labile subunit (ALS) to assess bioavailable rather than total IGF-1 [43,44]; and (iii) profile hepatic GHR pathway activity (e.g., GHR and SOCS2 expression) alongside metabolic readouts (insulin, NEFA). Notably, glucocorticoid-associated IGF-1 suppression in ruminants is context-dependent—varying with dose, duration, physiological stage, and nutrition [45,46].

Despite these advances, several limitations merit emphasis. The lack of direct, validated PTH measurements required reliance on UFEP as an indirect marker—informative but not a substitute for serum PTH. Species-specific features of IGF-1 regulation in sheep may limit direct translational generalization to humans [47]. Assay variability for DKK-1 and cathepsin-K underscores the need for species-specific validation, as these markers may differ across species [48]. For PTH, DKK-1, and sclerostin, we explicitly acknowledge fit-for-purpose limitations: absent ovine calibration, spike recovery, and parallelism preclude quantitative inference; results are, by design, descriptive [21,22,23]. To mitigate these constraints in future work, we will pursue targeted LC–MS/MS with stable-isotope dilution and/or immuno-MRM/SISCAPA as orthogonal methods to confirm concentrations and reduce epitope dependency [36,37,38,39].

Finally, the absence of a diet-only (non-OVX) arm and minor attrition (OVXDS n = 7 at 8 M) are acknowledged; our mixed-model approach accommodated occasional missingness and unequal group sizes.

## 5. Conclusions

This study highlights the profound metabolic consequences of dietary deficiency and glucocorticoid exposure in osteoporosis, emphasizing the critical role of nutritional and hormonal regulation in bone homeostasis. The findings suggest that biochemical markers could serve as valuable tools for monitoring osteoporosis progression, particularly in settings where imaging-based diagnostics are not feasible. By addressing key knowledge gaps in bone-energy metabolism interactions, this study contributes to ongoing efforts to develop more precise, non-invasive, and accessible diagnostic approaches for osteoporosis management. Future research should continue to explore biomarker optimization and targeted therapeutic interventions, ensuring that these findings are translated into improved clinical strategies for osteoporosis prevention and treatment.

## Data Availability

The raw data supporting the conclusions of this article will be made available by the authors on request.

## Supplementary Materials

The following supporting information can be downloaded at: https://www.mdpi.com/article/10.3390/nu17213353/s1, Table S1. Number of animals contributing data at each time point. Control: 8 throughout; OVX: 7 throughout; OVXD: 8 throughout; OVXDC: 8 at 0 M and 3 M, 7 at 8M due to attrition. Table S2. DXA outcomes by group and time (ROI: abdominal width, L1–L6). Table S3. 25-OH-vitamin D by group and time (descriptive) where the thesis reported specific means ± SD, those are entered; otherwise, descriptive notes indicate relative changes (e.g., ~38× lower vs. Control at 3M in OVXDC). Serum 25-OH Vitamin D (ng/mL), mean ± SD—by group and time. Table S4. Serum creatinine (µmol/L), mean ± SD (n). Table S5. Exploratory biomarkers with limited cross-reactivity (descriptive only).

## Abbreviations

The following abbreviations are used in this manuscript:

BAP	Bone-specific alkaline phosphatase
EIA	Enzyme immunoassays
ELISA	Enzyme-linked immunosorbent assay
FEP	Fractionated excretion of phosphate
IGF-1	Insulin-like growth factor
ND	Not determined (not calculable by the program)
NEFA	non-esterified fatty acids
NTX	Amino (N-)-terminal telopeptide type I collagen
OVX	The ovariectomy group
OVXD	The ovariectomy + diet group
OVXDS	Ovariectomy + diet + glucocorticoid administration
UFEP	Urine-fractionated excretion of phosphate
