# Supplementary material for: Metabolic Disruption in Osteoporotic Sheep: Evaluating Vitamin D Deficiency and Cortisone Effects via Biochemical Markers
Source: Nutrients. 2025 Oct 24;17(21):3353. doi: 10.3390/nu17213353 (PMC12610466; doi:10.3390/nu17213353)
Supplement: Supplementary file 1 [file nutrients-17-03353-s001.zip › nutrients-3923493-supplementary.pdf]

nutrients

Metabolic Disruption in Osteoporotic Sheep: Evaluating Vitamin D Deficiency and Cortisone Effects via Biochemical Markers

Gero Knapp [1,2,†], Judith Langenstein [3,†], Natali Bauer [3], Sabine Stötzel [2], Christian Heiss [1,2,4], Vahid Jahed [1], Muhammad Alzweiri [5], Christoph Biehl [1,2,†] and Thaqif El Khassawna [1,5,*,†]

[1] Experimental Trauma Surgery, Faculty of Medicine, Justus-Liebig-University of Giessen, Aulweg 128, 35392 Giessen, Germany; gero.knapp@chiru.med.uni-giessen.de (G.K.); christian.heiss@chiru.med.uni-giessen.de (C.H.); christoph.biehl@chiru.med.uni-giessen.de (C.B.)
[2] Department of Trauma-, Hand- and Reconstructive Surgery, Faculty of Medicine, Justus-Liebig-University of Giessen, Rudolf-Buchheim-Strasse 7, 35392 Giessen, Germany
[3] Department of Veterinary Clinical Sciences, Clinical Pathology and Clinical Pathophysiology, Faculty of Veterinary Medicine, Justus-Liebig-University of Giessen, Frankfurter-Strasse 126, 35392 Giessen, Germany
[4] Biruni University, Istanbul 34015, Türkiye
[5] School of Pharmacy, The University of Jordan, Amman 11942, Jordan
* Correspondence: thaqif.elkhassawna@chiru.med.uni-giessen.de
† These authors contributed equally to this work.

Table S1. Number of animals contributing data at each time point. Control: 8 throughout; OVX: 7 throughout; OVXD: 8 throughout; OVXDC: 8 at 0M and 3M, 7 at 8M due to attrition.

Group	0M (n)	3M (n)	8M (n)	Notes
**Control**	8	8	8	
**OVX**	7	7	7	
**OVXD**	8	8	8	
**OVXDC**	8	8	7	One animal was lost before the final time point

Table S2. DXA outcomes by group and time (ROI: abdominal width, L1–L6)

Group	Time	BMD (g/cm²), mean ± SD (n)	BMC (g), mean ± SD (n)	Z-score, mean ± SD (n)	%Δ BMD vs 0M, mean ± SD (n)
**Control**	0M	1.105 ± 0.089 (8)	69.262 ± 14.018 (8)	−0.255 ± 0.810 (8)	—
	3M	1.096 ± 0.130 (8)	78.975 ± 20.878 (8)	−0.344 ± 1.188 (8)	−1.1 ± 5.1 (8)
	8M	1.146 ± 0.111 (8)	79.138 ± 18.250 (8)	0.117 ± 1.013 (8)	+3.7 ± 6.5 (8)
**OVX**	0M	1.140 ± 0.115 (7)	77.914 ± 13.021 (7)	0.057 ± 1.053 (7)	—
	3M	1.079 ± 0.085 (7)	80.714 ± 3.870 (7)	−0.496 ± 0.781 (7)	−5.0 ± 5.8 (7)
	8M	1.113 ± 0.102 (7)	81.429 ± 10.843 (7)	−0.187 ± 0.935 (7)	−2.2 ± 5.1 (7)
**OVXD**	0M	1.138 ± 0.134 (8)	78.925 ± 14.050 (8)	0.039 ± 1.227 (8)	—
	3M	1.030 ± 0.096 (8)	69.912 ± 10.526 (8)	−0.942 ± 0.874 (8)	−9.0 ± 6.9 (8)
	8M	1.008 ± 0.123 (8)	72.412 ± 16.413 (8)	−1.143 ± 1.123 (8)	−11.2 ± 7.1 (8)
**OVXDS**	0M	1.152 ± 0.113 (8)	78.288 ± 14.686 (8)	0.166 ± 1.032 (8)	—
	3M	0.812 ± 0.083 (8)	49.500 ± 14.504 (8)	−2.939 ± 0.754 (8)	−29.3 ± 6.6 (8)
	8M	0.747 ± 0.054 (7)	35.371 ± 13.215 (7)	−3.531 ± 0.489 (7)	−34.4 ± 8.8 (7)

Table S3. 25-OH-vitamin D by group and time (descriptive) where the thesis reported specific means±SD, those are entered; otherwise, descriptive notes indicate relative changes (e.g., ~38× lower vs Control at 3M in OVXDC). Serum 25-OH Vitamin D (ng/mL), mean ± SD — by group and time

Group	0 M	3 M	8 M
**Control**	37.94 ± 11.80	N/A	23.38 ± 11.12
**OVX**	N/A	N/A	N/A
**OVXD**	30.00 ± 9.68	N/A	N/A
**OVXDC**	20.66 ± 4.74	~0 (38× lower vs Control)	4.84 ± 7.33

Notes. OVX values remained within the reference interval at all time points; numeric means not reported in source. OVXD 8M not significantly different from Control/OVX; numeric mean not reported. Reference interval (sheep): 8.14–55.71 ng/mL.

Table S4. Serum creatinine (µmol/L), mean ± SD (n)

Group	0 M	3 M
Control	—	55.0 ± 13.7 (n=8)
OVX	—	66.1 ± 14.0 (n=7)
OVXD	94.8 ± 9.8 (n=5)	73.8 ± 12.6 (n=8)
OVXDS	80.7 ± 12.9 (n=6)	64.6 ± 18.4 (n=7)

Table S5. Exploratory biomarkers with limited cross-reactivity (descriptive only)

Analyte	Platform (Kit)	Status in this study	Keynote (precision/cross-reactivity)
**PTH**	Human ELISA	Excluded	Near blank/non-reactive in ovine serum; not inferential.
**Sclerostin**	Human ELISA	Excluded	High variability, uncertain ovine cross-reactivity.
**Cathepsin-K**	Human ELISA	Excluded	Poor repeatability/unspecific binding.
**DKK-1**	Human ELISA	Descriptive only	Low but measurable; CV ≈ 5% (e.g., ~5.19 ± 0.25 pmol/L at 3M; ~4.70 ± 0.25 pmol/L at 8M).
